# Protein Analysis of Atrial Fibrosis via Label-Free Proteomics in Chronic Atrial Fibrillation Patients with Mitral Valve Disease

**DOI:** 10.1371/journal.pone.0060210

**Published:** 2013-04-04

**Authors:** Peide Zhang, Wei Wang, Xin Wang, Xu Wang, Yunhu Song, Yong Han, Jing Zhang, Hui Zhao

**Affiliations:** 1 Cardiovascular Surgery Department, Fuwai Hospital, Peking Union Medical College, Chinese Academy of Medical Sciences, Beijing, People’s Republic of China; 2 Department of Biochemistry and Molecular Biology, Peking University Cancer Hospital and Institute, Beijing, People’s Republic of China; Loyola University Chicago, United States of America

## Abstract

**Background:**

Atrial fibrosis, as a hallmark of atrial structure remodeling, plays an important role in maintenance of chronic atrial fibrillation, but interrelationship of atrial fibrosis and atrial fibrillation is uncertain. Label-free proteomics can implement high throughput screening for finding and analyzing pivotal proteins related to the disease.. Therefore, we used label-free proteomics to explore and analyze differentially proteins in chronic atrial fibrillation patients with mitral valve disease.

**Methods:**

Left and right atrial appendages obtained from patients with mitral valve disease were both in chronic atrial fibrillation (CAF, AF≥6 months, n = 6) and in sinus rhythm (SR, n = 6). One part of the sample was used for histological analysis and fibrosis quantification; other part were analyzed by label-free proteomic combining liquid chromatography with mass spectrometry (LC-MS), we utilized bioinformatics analysis to identify differential proteins.

**Results:**

Degree of atrial fibrosis was higher in CAF patients than that of SR patients. 223 differential proteins were detected between two groups. These proteins mainly had vital functions such as cell proliferation, stress response, focal adhesion apoptosis. We evaluated that serine/threonine protein kinase N2 (PKN2), dermatopontin(DP), S100 calcium binding protein B(S100B), protein tyrosine kinase 2(PTK2) and discoidin domain receptor tyrosine kinase 2(DDR2) played important roles in fibrotic process related to atrial fibrillation.

**Conclusion:**

The study presented differential proteins responsible for atrial fibrosis in chronic atrial fibrillation patients through label-free proteomic analysis. We assessed some vital proteins including their characters and roles. These findings may open up new realm for mechanism research of atrial fibrillation.

## Introduction

Atrial fibrillation (AF) remains the most encountered arrhythmia in clinical practice and major cause of morbidity and mortality. [1] AF tends to be secondary to organic heart diseases, which contribute to a total risk of hospitalization from 20% to 30% per year for patients with AF. [2] It has been reported that atrial fibrosis is a common feature of AF. [3] The increase in fibrosis has been shown to cause abnormal conduction through the atria, encompassing a plethora of potentially irreversible intracellular and extracellular processes, creating substrate for AF. [4], [5] However, complexity of atrial fibrosis associated with AF is not clear.

Modern molecular medicine is rapidly moving beyond functional genomics to proteomics. Proteomics-based studies focus on the interactions of multiple proteins and their roles as part of biological system rather than the structure and function of one single component. [6] Now we can utilize analytical proteomic techniques to evaluate global protein changes in diseased heart. Moreover, histological and immunohistochemical studies have found differences between the left atria (LA) and the right atria (RA) previously, [7] which might be caused partly by the fact that the LA is the source of initiation and maintenance of AF, the vast majority of thrombi can be identified within left atrial appendage (LAA). [8], [9] Therefore, the present study aimed to understand changes of proteins occurring in LA and RA of AF patients with mitral valve disease using label-free proteomic and provided evidences on relationship between atrial fibrosis and AF.

## Materials and Methods

### Patients

12 patients were enrolled in our study, they were divided into CAF group (n = 6, AF>6 months before surgery) and SR group (n = 6, without history of AF). LAA and RAA were obtained from the same patients as specimens at the time of the mitral valve surgery in Fuwai hospital. Patients in SR were screened to ensure that they had never experienced AF by direct questioning about symptoms suggestive of such and by the retrospective analysis of all 12-lead electrocardiograms during their entire preoperative review periods. Patients with familial paroxysmal atrial fibrillation, hyperthyreosis, chronic heart failure, sick sinus syndrome, pulmonary heart disease, cardiomyopathy, renal disease and secondary thoracotomy were excluded from the study. Routinely preoperative 2-dimensional color echocardiography and coronary catheterization were performed. Preoperative functional status was recorded with New York Heart Association (NYHA) classifications. All procedures involving using of human tissue were approved by Fuwai hospital Ethics Committee (approval NO2011-367, Fuwai hospital). Written informed consent was obtained from all patients recruited into the study.

### Histological Analysis

Every patient’s tissue was divided into two sections, one section was fixed by 10% formalin and paraffin-embedded tissue was cut into slices of about 4 um thickness. The slices were deparaffinaged by dimethyl benzene and soaked into a series of gradient concentration from 100 to 75% of alcohol. Preparations were stained with hematoxylin-eosin staining (HE) and Masson’s trichrome staining method according routine procedures [10].

### Quantification Analysis of Fibrosis

We chose four slices of Masson’s trichrome staining from every patient’s tissue as fibrosis quantification samples, five different fields of each slice were observed by Olympus BX51 optical microscope (magnification 400×). Endomysial collagen (between individual myocytes) was calculated as fibrosis per field, perimysial collagen was not included. The blue color represented fibrotic area, images were collected by QIMAGING MicroPublisher 5.0 R7V photomechanical system, semi quantitative analysis of fibrosis area was conducted with Image-Pro Plus 6.0 Image analytical system. Fibrosis scores = collagen fiber area/total view area × 100%.

### Label-free Proteomic Analysis

Total lysates from atrial appendages were reduced with 10 mM DTT at 37°C for 30 min and alkylated with 25 mM iodoacetamide in the dark at room temperature for 30 min. Proteins in the sample were resolved on a 10% SDS-PAGE gel (Figure S1) and stained with coomassie blue G250. The entire gel lane was cut into 32 pieces, each piece was excised into 1 mm gel pieces followed by in-gel digestion with 10 ng/µl of Trypsin (Promega) at 37°C overnight according to the manufacturer’s protocol. The resulting peptides were dried and reconstituted in 5% acetonitrile (AcN) and 1% formic acid, and desalted by a small tip column packed with C18 material according to the manufacturer’s protocol (1.9 µm C18 18 cm×75 ID nano silica capillary column Sino-America Proteomics, Beijing, China). The dried eluted peptides taken liquid chromatography-mass spectrometry (LC-MS) analysis (Platform: Water’s NanoAcquity UPLC coupled to Thermo LTQ Orbitrap Velos Mass Spectrometer), quantification is performed in conjunction with data normalization procedures and retention time correction. [11] The sequence information of differentially expressed peptides in two groups was obtained using database searching. (Database: ‘ftp://ftp.ncbi.nlm.nih.gov/refseq/H_sapiens/’, database search platform: SageN Research Sorcerer Enterprise with TurboSequest v4.0.3). Thework was finished in Beijing Proteome Research Center, China.

### Bioinformatic Analysis

We analyzed and compared differential proteins of LAA and RAA in CAF and SR patients respectively. Entrez Database was used to find corresponding Gene ID or Gene symbol according to reference sequence proteins number (http://www.ncbi.nlm.nih.gov/sites/gquery). Venn diagram was done with GeneVenn, [12] we used GATHER, [13] Cytoscape 2.8 [14] and GeneMANIA Cytoscape plugin [15] to analyze main enrichment functions and pathways of identified proteins. Furthermore, proteins interaction network was built with GeneMANIA Cytoscape plugin and GNC pro (http://gncpro.sabiosciences.com/gncpro/gncpro.php).

### Statistical Analysis

Statistical analysis was carried out by SPSS17.0. All values were expressed as means±S.D. Data between the two groups was evaluated by Student’s t test. P<0.05 was considered statistically significant.

## Results

### Patient Characteristics

The clinical characteristics of the cohorts were summarized in Table 1. Significant differences were present among the groups in left atrial diameter and left ventricle end-diastolic diameter, which were impossible to overcome because persistent and longtime AF, but the erection fraction of two groups were at the normal level, representing no heart function difference in two groups. Age as an individual risk factor of AF also showed remarkable distinction.

**Table 1:** Clinical characteristic of patients with CAF and patients with SR.

	CAF(n = 6)	SR(n = 6)	T value	P value
Age (years)	63.83±6.074	53.33±9.158	2.344	0.041*
Gender (male/female)	2/4	3/3		
Underlying cardiac diseases (n)				
MVD	2	2		
MVD/AVD	1	1		
MVD/TVD	2	1		
MVD/AVD/TVD	1	2		
NYHA (II/III)	2/4	3/3		
Cardiothoracic ratio	0.59±0.1	0.54±0.08	0.87	0.405
Left atrium (mm)	52.83±9.174	35.67±2.251	4.451	0.001*
PA pressure (mmHg)	35.7±20.66	28.4±9.17	0.791	0.447
EF (%)	60.83±2.04	62.33±5.13	−0.666	0.52
LVEDD (mm)	55.33±3.077	48.17±5.154	2.924	0.015*

CAF: chronic atrial fibrillation; SR: sinus rhythm; MVD: mitral valve disease; AVD: aortic valve disease; TVD: tricuspid valve disease; NYHA: New York Heart Association Classification; PA: pulmonary artery; EF: ejection fraction; LVEDD: left ventricle end-diastolic diameter.

* P<0.05.

### Histological Analysis Results

**Figure 1:**
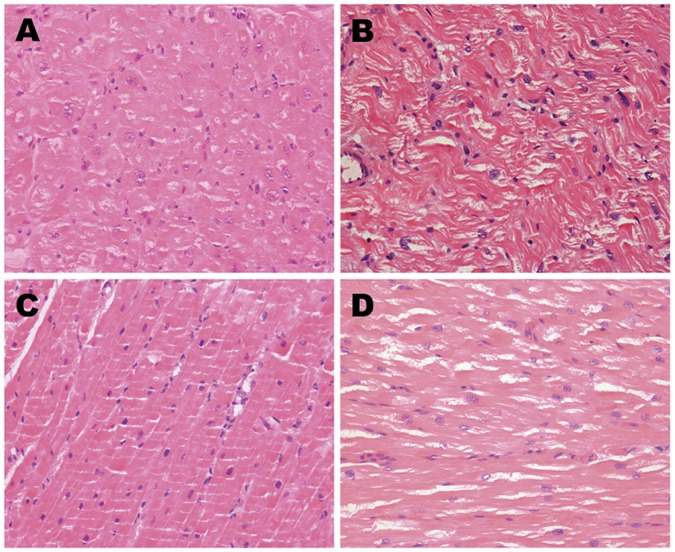
HE staining of LAA and RAA from CAF and SR patients. A. LAA of CAF patients; B. RAA of CAF patients; C. LAA of SR patients; D. RAA of SR patients.

**Figure 2:**
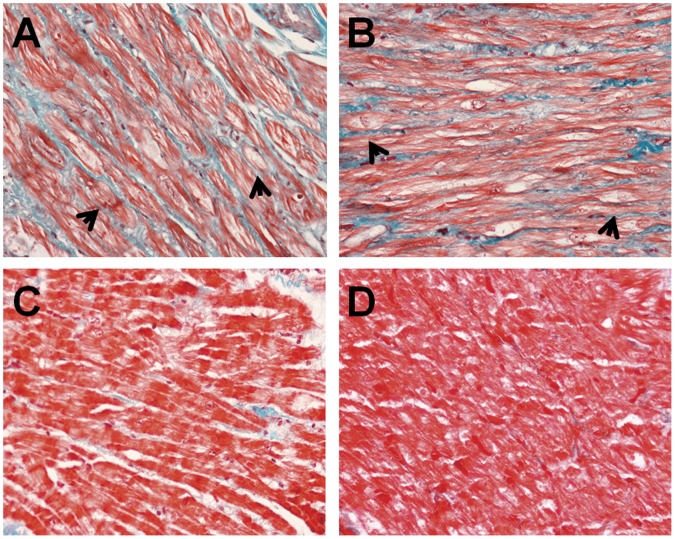
Masson’s trichrome staining of LAA and RAA from CAF and SR patients. A. LAA of CAF patients; B.RAA of CAF patients; C.LAA of SR patients; D.RAA of SR patients. (Arrows refer to myolysis).

Figure 1 and Figure 2 showed HE staining and Masson’s trichrome images. Both left and right atrial muscles in CAF group (Figure 1A, B) were observed to be divided into more branches and cell nucleus dispersed in disorder. In contrast, space between each branch was wider, than that in SR group that was orderly arrangement of atrial muscles and normal size nucleus (Figure 1C, D). In Masson’s trichrome images, the vast majority of myocytes were of uniform size and regularly organized within the trabeculae (Figure 2C, D) were observed in SR group. In CAF group, myocardial tissue showed large areas of myocytes with extensive myolysis, bundles of myofibers were separated by thick layers of fibrosis to form collagenous septa (Figure 2A, B). Furthermore, fibrotic degree seemed more severe in LAA than that in RAA of CAF patients.

### Results of Fibrosis Quantification

Quantification of fibrosis area was based on chosen views of Masson’s trichrome staining slices from every patient. Comparison between CAF and SR patients was shown in Table 2, fibrosis area was obvious in atrial appendages of CAF patients, LAA of CAF patients had the most big fibrosis area (9.54±3.06),there was statistically significance in comparison of LAA and RAA between the two groups (p<0.05). Fibrotic degree of LAA and RAA of CAF patients was approximately equal.

**Table 2:** Quantification of fibrosis area.

	Fibrosis area (%)	T value	P value
LAA-CAF	9.54±3.06		
RAA- CAF	7.59±2.0 8		
LAA- SR	4.27±1.12		
RAA - SR	4.81±1.15		
LAA-CAF/LAA-SR		3.969	0.003*
RAA-CAF/RAA-SR		2.862	0.017*
LAA-CAF/RAA-CAF		1.293	0.225

LAA-CAF: left atrial appendage in permanent atrial fibrillation; RAA-CAF: right atrial appendage in permanent atrial fibrillation; LAA-SR: left atrial appendage in sinus rhythm; RAA-SR: right atrial appendage in sinus rhythm.

* P<0.05.

### Proteins Analysis Results

**Figure 3:**
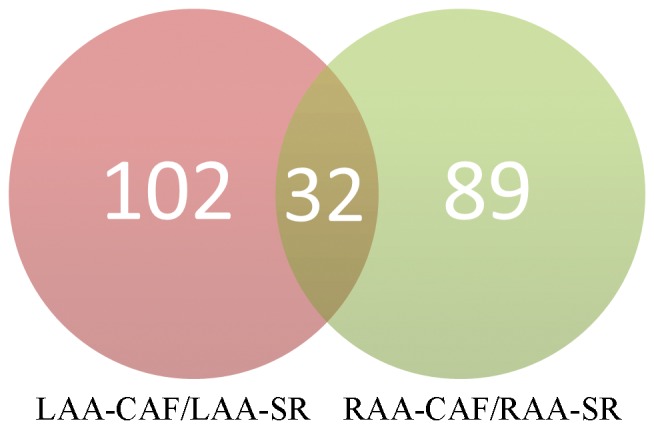
The Venn diagram shows identified differential proteins of two groups and their relationship.

**Figure 4:**
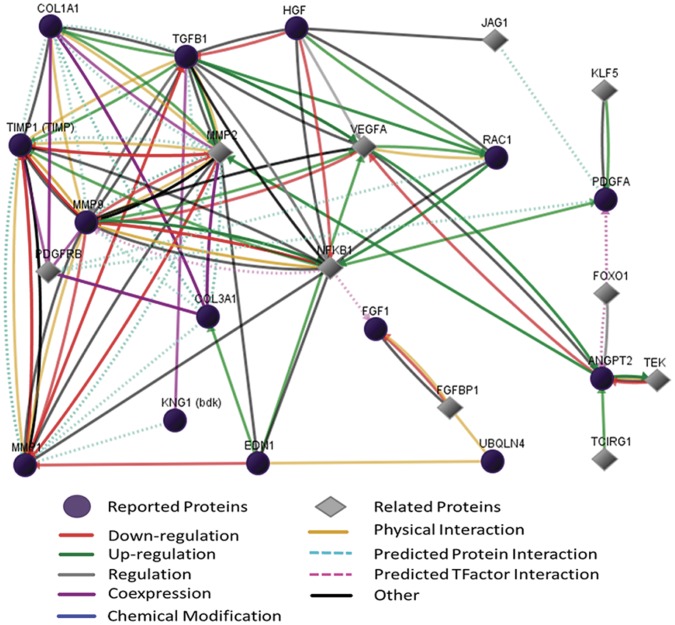
The network of reported proteins associated with atrial fibrosis. proteins names corresponded to gene symbol:COL1A1: collagen I; COL3A1: collagen III; TGFβ1:Transforming Growth factor β1; HGF:hepatocyte growth factor; TIMP:tissue inhibitor of metalloproteinases; MMP:matrix metallopeptidase; KNG1 (bdk):bradykinin (BK); EDN1:endothelin 1 (ET1); VEGFA:vascular endothelial growth factor A (VEGFa); RAC1:ras-related C3 botulinum toxin substrate 1(Rac1); NFKB:nuclear factor κB (NFκB); PDGFA:platelet-derived growth factor alpha polypeptide (PDGFa); FGF1:fibroblast growth factor 1; UBQLN4:connexin43; ANGPT2: angiopoietin 2 (Ang II); JAG1:protein jagged-1; FOXO1:forkhead box protein O1.

**Figure 5:**
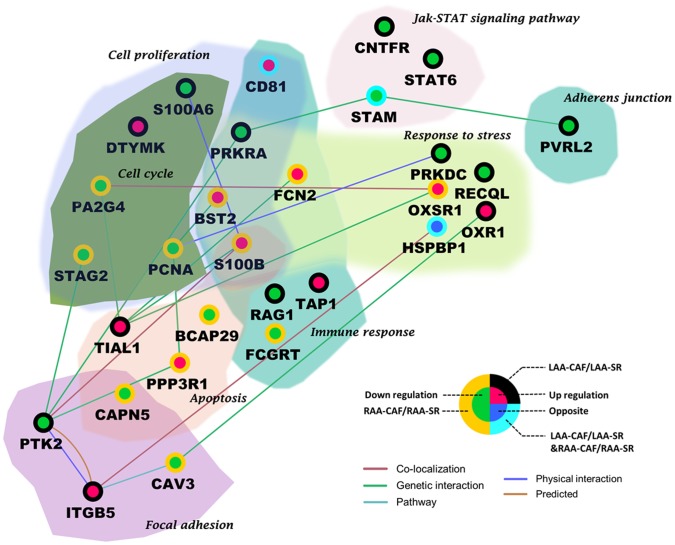
Functions and pathways of differential proteins. (Enrichment p-value<0.05). Protein names corresponded to gene symbols: S100A6:S100 calcium binding protein A6; DTYMK:deoxythymidylatekinase; PA2G4:proliferation-associated 2G4; STAG2:stromal antigen 2; PCNA:proliferating cell nuclear antigen; PRKRA: interferon-inducible double stranded RNA dependent activator; FCN2:ficolin 2; BST2:bone marrow stromal cell antigen 2; S100B:S100 calcium binding protein B; TIAL1:TIA1 cytotoxic granule-associated RNA binding protein-like 1; BCAP29:B-cell receptor-associated protein 29; RAG1:recombination activating gene 1; TAP1:transporter 1, ATP-binding cassette; FCGRT:Fc fragment of IgG receptor; PPP3R1:protein phosphatase 3, regulatory subunit B; CAPN5 :calpain 5; PTK2:protein tyrosine kinase 2; ITGB5:integrin, beta 5; CAV3:caveolin 3; CNTFR:ciliary neurotrophic factor receptor; STAT6:signal transducer and activator of transcription 6; STAM:signal transducing adaptor molecule; PRKDC:protein kinase, DNA-activated, catalytic polypeptide; RECQL:RecQ protein-like; OXSR1:oxidative-stress responsive 1; OXR1:oxidation resistance 1; HSPBP1:heat shock 70 binding protein, cytoplasmic cochaperone 1; PVRL2: poliovirus receptor-related 2.

**Figure 6:**
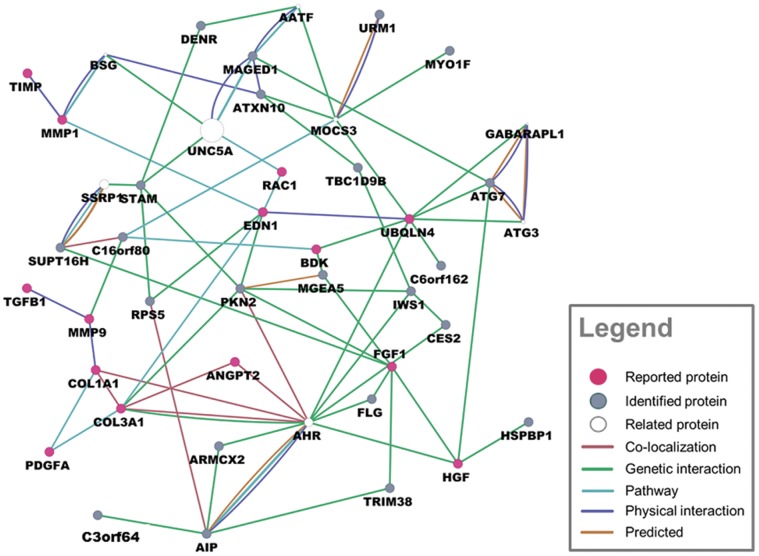
The relationship network of 32 common proteins and reported proteins. The size of the white ball means the association weight of relatedproteins and proteins in our dataset (reported proteinsand identified proteins). Proteins names corresponded to gene symbol refer to Table 3.C and Figure 4.

223 differential proteins were identified totally in two groups by label-free proteomic (p<0.05, Table S1,S2), every identified protein had four-fold change at least in both groups. In LAA comparison between CAF and SR patients, 134 differential proteins were found; in RAA comparison between CAF and SR, 121 differential proteins were found; 32 proteins were overlapping in two groups (Figure 3). Top 10 proteins according to p-value and overlapping proteins are shown in Table 3. In addition, many previous studies had reported several important proteins related to fibrotic process or atrial fibrillation like transforming Growth factor β1(TGFβ1), hepatocyte growth factor (HGF), tissue inhibitor of metalloproteinases (TIMP), these reported proteins were summarized in Figure 4. We also analyzed functions and pathways of differential proteins according to enrichment p-valve (p<0.05, Table S3). These functions and pathways included cell proliferation, cell cycle, apoptosis, focal adhesion, immune response, response to stress, adherent junction and JAK-STAT signaling pathway (Figure 5). Some proteins had more than one function such as S100 calcium binding protein B(S100B), calpain 5(CAPM5), bone marrow stromal cell antigen 2(BST2), proliferating cell nuclear antigen(PCNA). Finally we specially analyzed the interaction of 32 overlapping proteins with reported proteins (Figure 6).

**Table 3:** Top 10 and overlapped differential proteins of two groups.

Refseq	Gene ID	Gene symbol	Protein Description	Log2ratio	P value
**A:LAA-CAF/LAA-SR**					
NP_055054.1	8892	EIF2B2	translation initiation factor eIF-2B subunit beta	7.9	2.56E−12
NP_001601.1	53	ACP2	lysosomal acid phosphatase isoform 1 precursor	−7.86	3.17E−12
NP_061039.3	55435	AP1AR	AP-1 complex-associated regulatory protein isoform a	−7.38	6.17E−11
NP_037368.1	25814	ATXN10	ataxin-10 isoform 1	−6.12	5.77E−08
NP_036602.1	23534	TNPO3	transportin-3 isoform 1	6.03	9.04E−08
NP_115716.1	84300	C6orf125	hypothetical protein LOC84300	5.52	9.86E−07
NP_006535.1	10640	EXOC5	exocyst complex component 5	−5.49	1.12E−06
NP_055830.1	23030	KDM4B	lysine-specific demethylase 4B	5.01	8.93E−06
NP_004328.1	734	OSGIN2	oxidative stress-induced growth inhibitor 2 isoform 2	−4.99	9.75E−06
NP_001098.1	97	ACYP1	acylphosphatase-1 isoform a	4.91	1.35E−05
**B:RAA-CAF/RAA-SR**					
NP_078970.3	79747	C6orf103	calpain-7-like protein	7.51	8.13E−11
NP_000584.2	6890	TAP1	antigen peptide transporter 1	−7.14	6.20E−10
NP_001139186.1	10055	SAE1	SUMO-activating enzyme subunit 1 isoform c	−6.77	4.59E−09
NP_004347.1	975	CD81	CD81 antigen	−6.38	3.33E−08
NP_004328.1	734	OSGIN2	oxidative stress-induced growth inhibitor 2 isoform 2	−6.36	3.65E−08
NP_060264.3	54914	KIAA1797	hypothetical protein LOC54914	5.75	6.36E−07
NP_065916.2	170506	DHX36	probable ATP-dependent RNA helicase DHX36 isoform 1	−5.71	7.61E−07
NP_079517.1	80700	UBXN6	UBX domain-containing protein 6 isoform 1	5.67	9.06E−07
NP_061956.2	54482	CCDC76	tRNA guanosine-2′-O-methyltransferase TRM13 homolog	−5.56	1.51E−06
NP_036399.3	23640	HSPBP1	hsp70-binding protein 1	5.5	1.94E−06
**C: overlapping proteins of two groups**					
NP_037368.1	25814	ATXN10	ataxin-10 isoform 1		
NP_004328.1	734	OSGIN2	oxidative stress-induced growth inhibitor 2 isoform 2		
NP_112176.1	81605	URM1	ubiquitin-related modifier 1 homolog isoform a		
NP_775925.1	285203	C3orf64	AER61 glycosyltransferase		
NP_003767.2	64976	MRPL40	39S ribosomal protein L40, mitochondrial precursor		
NP_003860.2	8824	CES2	cocaine esterase isoform 1		
NP_036399.3	23640	HSPBP1	hsp70-binding protein 1		
NP_055597.1	9823	ARMCX2	armadillo repeat-containing X-linked protein 2		
NP_001000.2	6193	RPS5	40S ribosomal protein S5		
NP_005773.3	10189	THOC4	THO complex subunit 4		
NP_689979.1	254863	C17orf61	hypothetical protein LOC254863 precursor		
NP_006247.1	5586	PKN2	serine/threonine-protein kinase N2		
NP_065158.3	57150	C6orf162	hypothetical protein LOC57150		
NP_037374.1	29105	C16orf80	transcription factor IIB		
NP_000230.1	4069	LYZ	lysozyme C precursor		
NP_004347.1	975	CD81	CD81 antigen		
NP_001185987.1	1E+08	C15orf38-AP3S2	C15orf38-AP3S2 fusion protein		
NP_001135906.1	10724	MGEA5	bifunctional protein NCOAT isoform b		
NP_078970.3	79747	C6orf103	calpain-7-like protein		
NP_006346.1	10475	TRIM38	tripartite motif-containing protein 38		
NP_003464.1	8027	STAM	signal transducing adapter molecule 1		
NP_060439.2	55677	IWS1	protein IWS1 homolog		
NP_002007.1	2312	FLG	filaggrin		
NP_036467.2	4542	MYO1F	myosin-If		

RefSeq: reference sequence; Log2ratio: positive value shows up-regulation, negative value shows down-regulation. P<0.05.

## Discussion

Until now, there is no explicit mechanism responsible for AF. It has been demonstrated that electrical, contractile and structural remodeling contribute to AF occurrence and maintenance in both animal models and in patients. [16] Fibrosis as the hallmark of atrial structural remodeling results from an accumulation of collagen to replace degenerating myocytes. [17] Fibrotic alteration in atria structure may destroy normal electrical conduction and promote formation of ectopic pacemaker, which intervenes normal pace making through breaking junction among myocytes and cardiocytes apoptosis. [18] All these changes provide suitable environment for AF occurrence and maintenance. The present study demonstrated mostly proteins variations were connected with fibrotic process, some differential proteins may have impacts on ectopic pace making and abnormal conduction. On the other hand, theoretical models have implicated atrial interstitial fibrosis is a basic mechanism and an endpoint for AF, [19] In our study, over production of collagens disturbed myocytes continuity (Figure 1A, B) in CAF patients, leading to myocytes degeneration and extensive myolysis (Figure 2A, B), fibrosis degree was much higher in patients with CAF(Table 2)The finding was in accordance with past research that revealed myolysis and interstitial fibrosis were consequences of AF. [20] As explained above we induced that atrial fibrosis and CAF were interrelated. Although all patients had mitral valve disease, patients with CAF appeared larger left atrial diameter and left ventricle end-diastolic diameter because of hemodynamic instability resulted from atrial fibrillation for long time (Table 1), and we excluded the proteins associated with atria dilatation [21]. Moreover, age as an independent risk factor was inevitable in patient selection, [22] but differential proteins participating in aging process could be ignored in the same person and excluded from proteomic screening. LAA and RAA of CAF patients also showed inconsistent fibrotic degree, which might be attributed to approaching 200 differential proteins identified by label-free proteomic, and this obvious change on proteins could help researchers to investigate the reason why LA was the source of AF.

Previous studies had found that some proteins played important roles in atrial fibrosis related to AF. We reviewed these reported proteins and analyzed their intimate relationships (Figure 4). Transforming growth factor β1 (TGFβ1) was an key factor to promote myofibroblasts differentiation, [23] it was induced by Angiotensin II (Ang II) and stimulated matrix metallopeptidase 2 (MMP2) to increase collagen production through SMAD signaling pathway, [24], [25] it also resulted in connexin43 remodeling, a vital factor for connection between cardiomyocytes. [26] Similarly, Chia-ti Tsai et al found Ang II/Rac1/STAT signaling pathway participates in atrial structural remodeling. [27] Moreover, platelet-derived growth factor alpha polypeptide (PDGFa) stimulated cell proliferation, migration, differentiation and physiological function of mesenchymal cells, some data showed that atrial fibrosis and AF susceptibility was promoted by inflammation via PDGF related mechanisms. [28] In addition, hepatocyte growth factor (HGF) could inhabit fibrotic development by depressing TGFβ1, [29] fibroblast growth factor 1(FGF1) produced myofibroblasts dedifferentiation, [30] nuclear factor κB(NF-κB) and vascular endothelial growth factor A (VEGFa) connected tightly with many proteins above mentioned, but they were not discussed here.

We identified 223 differential proteins in all by label-free proteomic technique, which possesses higher proteome coverage and peptide scores than isotope label method, permitting fast quantitative profiling of a large number of proteins from complex biological samples. These differential proteins, connecting illustrated changes in fibrotic process related to CAF (Figure 5), thus atrial remodeling was outcome of cellular synthesized biological activity rather than over deposition of fibrillar collagen in extracellular matrix only. Here we introduced some functions and biological processes. Fibroblast proliferation, which established cell to cell contacted in myolysis and dedifferentiation myocytes, played a key role in maintaining the normal structure and function of atrial myocytes [20], our study showed down-regulation of PCNA, S1006A, PA2G4 and STAG2could accelerate collagen increase, which affected myofibroblasts differentiation and unbalanced the ratio of collagen types. S.Eiras reported atrial fibrosis may destroy normal joint between cardiomyocytes, making atrial contractile function unstable, the stress to atria might be related to atrial dilatation. [31] Response to stress involved proteins such as PRKDC, RECQL and HSPBP1 might participate in this process and connect atrial fibrosis with dilatation. Immune response reflected inflammation effect on CAF patients. RAG1, TAP1, FCGRT and CD81 were identified, these proteins were consistent with Alex Y.Tan’s study that some inflammation factors were relevant to AF. [32] However, some of patients had rheumatic mitral valve diseases, so it was difficult to judge which factor caused the immune response. The Janus Kinase and Signal Transducer and Activator of Transcription pathway (JAK-STAT pathway) had been connected with fundamental cellular signaling events such as innate and adaptive immune responses, cell growth and apoptosis process regulation, [33] the pathway had the dual function of both transducing signals from receptors at the cell surface to the nucleus and activating transcription of target genes in the nucleus of cells, [34] differential proteins such as CNTFR, STAM and STAT6 involved in the pathway and were closely bound to catalytic activity,transcription regulation and apoptosis process.

There were 32 proteins were overlapped in 223 differential proteins, these proteins might engage in some routine procedures of fibrotic process on both atriums. We analyzed connection of 32 proteins with reported proteins (Figure 6), they interacted closely, we took serine/threonine protein kinase N2 (PKN2) for example. PKN2 was one of three serine/threonine kinases targeting to Rho family GTPases. It could be activated by PDGF to disturb cell to cell adhesion and control cellular activities such as cell cycle, [35], [36] which was key processes of atrial fibrosis. Similarly, other overlapped proteins were also important in fibrotic course. On the other hand, there were 191 different identified proteins in two comparison groups except for 32 overlapped proteins. The distinction might expain the different ability of RAA and LAA to adapt to AF at molecular level. Protein expression and remodeling process might affect ectopic beats origin and autonomic ganglia distributed in the pulmonary veins, [37], [38] but the mechanism still uncertain Some representative differential proteins were discussed here. In LAA comparing group, dermatopontin (DP) and S100 calcium binding protein B (S100B) contacted with reported proteins closely (Figure S2). DP was small molecular weight noncollagenous component in extracellular matrix. First been described in 1989, [39] DP expressed strongly around the infarct zone of an experimental myocardial infarction model, regulating diameter and architecture of collagen fibrils by interacting with collagen molecules for preventing abnormal fibril assembly. [40] Osamu Okamoto found DP increased the cellular response to TGFβ and regulated biological activity when up-regulated. [41] We deduced DP might be involved in tissue fibrosis; S100B was member of S100 family, expressing mainly in astrocytes, which correlated central nervous system injury and cardiac lesion after cardiac surgeries or cardiac dysfunction. [42], [43], [44] It also could regulate fibroblast growth factor 1(FGF1) and PDGFa in our analysis, thus S100B might act as key factor on repairing and adaptation to tissue damage and remodeling. In RAA comparing group, protein tyrosine kinase 2 (PTK2) and discoidin domain receptor tyrosine kinase 2 (DDR2) were analyzed (Figure S3). PTK2 (alias focal adhesion kinase, FAK) was a member of the focal adhesion kinases subfamily, it controlled cell immigration, proliferation and survival through integrin regulating signaling. We found PTK2 could result in myofibroblasts differentiation through TGFβ1 and linked with HGF, MMP9 and RAC1, which were important factors in atrial fibrosis with AF. [45] DDR2 was member of receptor tyrosine kinases, fibrillar collagens selectively stimulated DDR2 as ligand. Vogel.W found prolonged activation of DDR2 by collagen was associated with an up-regulation of matrix metalloprotease-1 (MMP1), an enzyme that specifically decomposed native fibrillar collagen. [46] Besides, DDR2 also were found to connect with PDGFa and Ang II by MMP2. We deduced that major function of DDRs was to monitor the formation of collagenous extracellular matrix by regulating the synthesis of collagens and their degrading enzymes.

### Study Limitations

Our main limitation is the small number of patients, partly because of restriction of patients selected in this study. Patients with sole mitral valve disease in sinus rhythm were in minority due to permanent unstable hemodynamic, most of patients combined with other underlying disease such as coronary heart disease, pulmonary hypertension, heart failure. In addition, we just investigated that differential proteins associated with atrial fibrotic process were consequences of CAF with mitral valve because of enrolled CAF patients, we could not explain atrial fibrosis as one cause of CAF in our study, but these differential proteins must have impact on AF maintenance, further studies would be needed. The other limitation was the present study only provided description of the changes in atrial fibrosis at protein level, risk factor of AF including age, left atrial diameter and left ventricle end-diastolic diameter might influence proteins alteration of atrial fibrosis, we needed to heed the interrelationship of these risk factors of CAF.

### Conclusion

Label-free proteomic provides a way to study atrial fibrosis related to atrial fibrillation comprehensively at molecular level. We found 223 differential proteins were closely connected with atrial fibrosis in CAF patients with mitral valve disease and described involved functions and pathways. These differential proteins demonstrated the potential relation between atrial fibrosis and CAF, and it was important to explore mechanisms on structural remodeling of CAF. In conclusion, the full view of differential proteins via label-free proteomic in atrial appendages of CAF patients may open up new realm for mechanism research of atrial fibrillation.

## Supporting Information

Figure S1 **10% SDS-PAGE gel diagram.** Lysates are from atrial appendages from permanent atrial fibrillation and sinus rhythm patients. Compound of each group was composed of 6 samples.LAA-CAF:left atrial appendage in permanent atrial fibrillation; RAA-CAF: right atrial appendage in permanent atrial fibrillation; LAA-SR: left atrial appendage in sinus rhythm; RAA-SR: right atrial appendage in sinus rhythm.
(TIF)

Figure S2 **Interaction network between LAA differential proteins and reported proteins.** (Arrow shows described proteins in the article).
(TIF)

Figure S3 **Interaction network between RAA differential proteins and reported proteins.** (Arrow shows described proteins in the article).
(TIF)

Table S1 **102 differential proteins in LAA of CAF and SR patients.** RefSeq: reference sequence; Log2ratio: positive value shows up-regulation, negative value shows down-regulation. P<0.05.
(DOC)

Table S2 **89 differential proteins in RAA of CAF and SR patients.** RefSeq: reference sequence; Log2ratio: positive value shows up-regulation, negative value shows down-regulation. P<0.05.
(DOC)

Table S3 **Enrichment p-valve in functions and pathways of 223 differentially expressed proteins identified by Label-free proteomic (p<0.05).**
(DOC)
