# Supplementary figures and images for: Protein Analysis of Atrial Fibrosis via Label-Free Proteomics in Chronic Atrial Fibrillation Patients with Mitral Valve Disease
Source: PLoS One. 2013 Apr 4;8(4):e60210. doi: 10.1371/journal.pone.0060210 (PMC3617171; doi:10.1371/journal.pone.0060210)

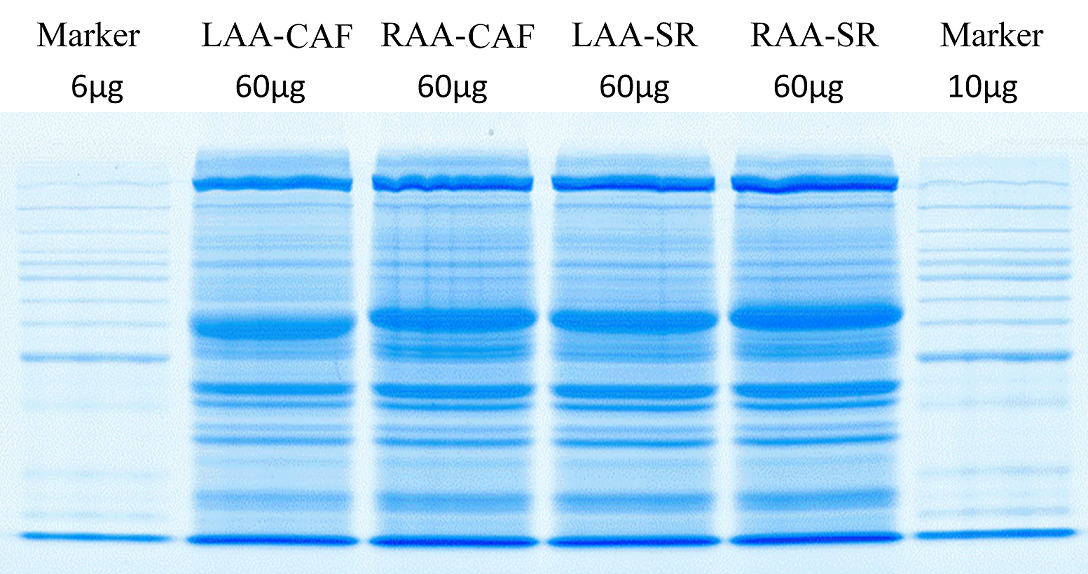

Supplement: Figure S1 — 10% SDS-PAGE gel diagram. Lysates are from atrial appendages from permanent atrial fibrillation and sinus rhythm patients. Compound of each group was composed of 6 samples.LAA-CAF:left atrial appendage in permanent atrial fibrillation; RAA-CAF: right atrial appendage in permanent atrial fibrillation; LAA-SR: left atrial appendage in sinus rhythm; RAA-SR: right atrial appendage in sinus rhythm. (TIF) [file pone.0060210.s001.tif]

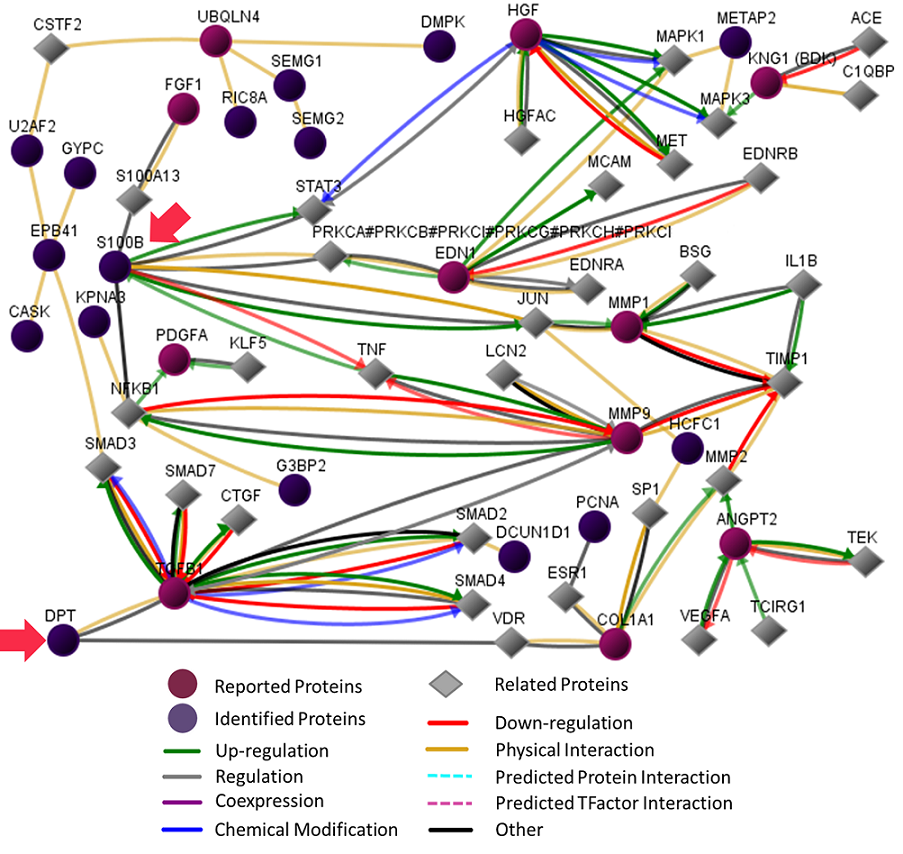

Supplement: Figure S2 — Interaction network between LAA differential proteins and reported proteins. (Arrow shows described proteins in the article). (TIF) [file pone.0060210.s002.tif]

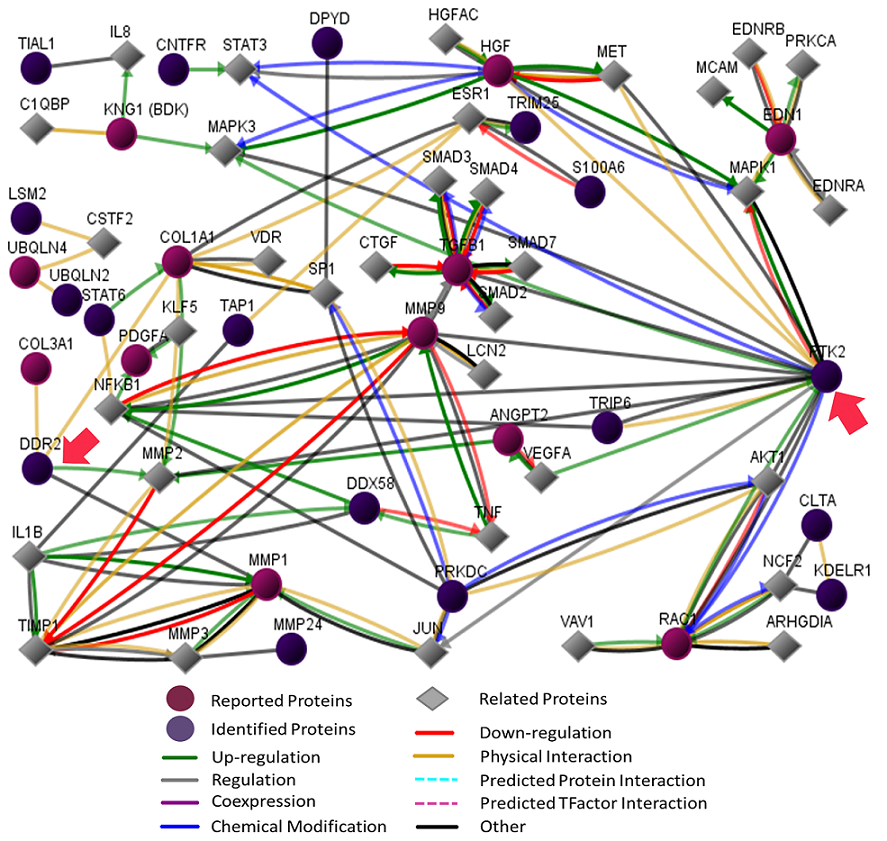

Supplement: Figure S3 — Interaction network between RAA differential proteins and reported proteins. (Arrow shows described proteins in the article). (TIF) [file pone.0060210.s003.tif]
