# Supplementary material for: Protein Analysis of Atrial Fibrosis via Label-Free Proteomics in Chronic Atrial Fibrillation Patients with Mitral Valve Disease
Source: PLoS One. 2013 Apr 4;8(4):e60210. doi: 10.1371/journal.pone.0060210 (PMC3617171; doi:10.1371/journal.pone.0060210)
Supplement: Table S1 — 102 differential proteins in LAA of CAF and SR patients. RefSeq: reference sequence; Log2ratio: positive value shows up-regulation, negative value shows down-regulation. P<0.05. (DOC) [file pone.0060210.s004.doc]

**Table S1 102 differential proteins in LAA of CAF and SR patients**

| Refseq | Description | Log2ratio CAF/SR | p-Val CAF/SR |
| --- | --- | --- | --- |
| NP_055054.1 | translation initiation factor eIF-2B subunit beta | 7.9 | 2.56E-12 |
| NP_001601.1 | lysosomal acid phosphatase isoform 1 precursor | -7.86 | 3.17E-12 |
| NP_061039.3 | AP-1 complex-associated regulatory protein isoform a | -7.38 | 6.17E-11 |
| NP_037368.1 | ataxin-10 isoform 1 | -6.12 | 5.77E-08 |
| NP_036602.1 | transportin-3 isoform 1 | 6.03 | 9.04E-08 |
| NP_115716.1 | hypothetical protein LOC84300 | 5.52 | 9.86E-07 |
| NP_006535.1 | exocyst complex component 5 | -5.49 | 1.12E-06 |
| NP_055830.1 | lysine-specific demethylase 4B | 5.01 | 8.93E-06 |
| NP_004328.1 | oxidative stress-induced growth inhibitor 2 isoform 2 | -4.99 | 9.75E-06 |
| NP_001098.1 | acylphosphatase-1 isoform a | 4.91 | 1.35E-05 |
| NP_004099.2 | ficolin-2 isoform a precursor | -4.83 | 1.84E-05 |
| NP_115550.2 | mitochondrial import receptor subunit TOM40B | -4.77 | 2.35E-05 |
| NP_112176.1 | ubiquitin-related modifier 1 homolog isoform a | 4.59 | 4.68E-05 |
| NP_775925.1 | AER61 glycosyltransferase | 4.55 | 5.60E-05 |
| NP_612638.3 | histidine triad nucleotide-binding protein 3 | -4.54 | 5.81E-05 |
| NP_987101.1 | ras GTPase-activating protein-binding protein 2 isoform a | 4.54 | 5.83E-05 |
| NP_219500.1 | transmembrane protein C9orf123 | 4.53 | 5.97E-05 |
| NP_003767.2 | 39S ribosomal protein L40, mitochondrial precursor | 4.4 | 9.63E-05 |
| NP_003860.2 | cocaine esterase isoform 1 | 4.36 | 1.12E-04 |
| NP_002258.2 | importin subunit alpha-3 | 4.11 | 1.18E-04 |
| NP_036399.3 | hsp70-binding protein 1 | -4.33 | 1.24E-04 |
| NP_115682.1 | probable alpha-ketoglutarate-dependent dioxygenase ABH7 precursor | -4.32 | 1.28E-04 |
| NP_002998.1 | semenogelin-1 preproprotein | -4.25 | 1.36E-04 |
| NP_068751.4 | synembryn-A | -4.1 | 2.80E-04 |
| NP_904325.2 | kinesin-like protein KIF1B isoform alpha | 4.1 | 2.84E-04 |
| NP_055597.1 | armadillo repeat-containing X-linked protein 2 | -4.08 | 2.99E-04 |
| NP_001000.2 | 40S ribosomal protein S5 | 4.07 | 3.09E-04 |
| NP_005773.3 | THO complex subunit 4 | 4.03 | 3.53E-04 |
| NP_001191344.1 | tubulointerstitial nephritis antigen-like isoform 3 | 4.03 | 3.60E-04 |
| NP_689979.1 | hypothetical protein LOC254863 precursor | 3.78 | 3.88E-04 |
| NP_203123.1 | caveolin-3 | 3.91 | 5.27E-04 |
| NP_001011671.1 | coiled-coil-helix-coiled-coil-helix domain-containing protein 7 isoform f | 3.86 | 6.20E-04 |
| NP_006247.1 | serine/threonine-protein kinase N2 | -3.82 | 7.13E-04 |
| NP_065158.3 | hypothetical protein LOC57150 | -3.79 | 7.74E-04 |
| NP_003869.1 | gamma-glutamyl hydrolase precursor | -3.75 | 8.95E-04 |
| NP_078986.1 | isochorismatase domain-containing protein 2, mitochondrial isoform 2 | 3.52 | 9.07E-04 |
| NP_001138587.1 | putative protein phosphatase 1 regulatory inhibitor subunit 3G | -3.71 | 1.02E-03 |
| NP_976218.1 | protein 4.1 isoform 2 | -3.7 | 1.05E-03 |
| NP_002092.1 | glycophorin-C isoform 1 | -3.66 | 1.18E-03 |
| NP_061917.3 | coiled-coil domain-containing protein 93 | -3.65 | 1.21E-03 |
| NP_001171821.1 | FAD synthase isoform 4 | -3.64 | 1.23E-03 |
| NP_002999.1 | semenogelin-2 precursor | -3.62 | 1.34E-03 |
| NP_001008405.1 | B-cell receptor-associated protein 29 isoform a | 3.62 | 1.35E-03 |
| NP_061885.2 | pleckstrin homology domain-containing family A member 5 isoform 1 | 3.55 | 1.66E-03 |
| NP_037374.1 | transcription factor IIB | 3.54 | 1.71E-03 |
| NP_001928.2 | dermatopontin precursor | 3.5 | 1.91E-03 |
| NP_006003.1 | putative ATP-dependent Clp protease proteolytic subunit, mitochondrial precursor | -3.49 | 1.97E-03 |
| NP_005325.2 | host cell factor 1 | -3.49 | 1.98E-03 |
| NP_067013.1 | polypyrimidine tract-binding protein 2 | -3.48 | 2.05E-03 |
| NP_002583.1 | proliferating cell nuclear antigen | 3.46 | 2.17E-03 |
| NP_068766.1 | serine racemase | -3.44 | 2.26E-03 |
| NP_061119.1 | core histone macro-H2A.2 | 3.44 | 2.30E-03 |
| NP_057090.2 | 1-acylglycerol-3-phosphate O-acyltransferase ABHD5 | 3.43 | 2.37E-03 |
| NP_078941.2 | F-box-like/WD repeat-containing protein TBL1XR1 | -3.41 | 2.51E-03 |
| NP_000230.1 | lysozyme C precursor | -3.4 | 2.55E-03 |
| NP_004347.1 | CD81 antigen | -3.4 | 2.58E-03 |
| NP_001031.2 | sex hormone-binding globulin isoform 1 precursor | -3.32 | 3.26E-03 |
| NP_001185987.1 | C15orf38-AP3S2 fusion protein | -3.29 | 3.57E-03 |
| NP_001135906.1 | bifunctional protein NCOAT isoform b | 3.27 | 3.74E-03 |
| NP_078970.3 | calpain-7-like protein | 3.27 | 3.75E-03 |
| NP_001075031.1 | myotonin-protein kinase isoform 4 | -3.26 | 3.90E-03 |
| NP_057000.2 | calcium homeostasis modulator protein 2 | 3.22 | 4.26E-03 |
| NP_006346.1 | tripartite motif-containing protein 38 | 3.19 | 4.66E-03 |
| NP_001014364.1 | filaggrin-2 | -3.19 | 4.74E-03 |
| NP_003931.2 | ubiquitin carboxyl-terminal hydrolase 13 | -3.24 | 4.81E-03 |
| NP_056208.2 | prostate androgen-regulated mucin-like protein 1 precursor | -3.15 | 5.28E-03 |
| NP_055971.1 | REST corepressor 1 | 3.14 | 5.34E-03 |
| NP_003464.1 | signal transducing adapter molecule 1 | 2.93 | 6.37E-03 |
| NP_003236.3 | protein-glutamine gamma-glutamyltransferase E precursor | -3.08 | 6.40E-03 |
| NP_060439.2 | protein IWS1 homolog | 3.07 | 6.49E-03 |
| NP_065189.1 | chronic lymphocytic leukemia deletion region gene 6 protein isoform 1 | 3.04 | 6.96E-03 |
| NP_002007.1 | filaggrin | -3.03 | 7.15E-03 |
| XP_003119797.1 | PREDICTED: putative uncharacterized serine/threonine-protein kinase SgK110-like | -3.02 | 7.36E-03 |
| NP_001119526.1 | peripheral plasma membrane protein CASK isoform 2 | -3.01 | 7.57E-03 |
| NP_004046.2 | calpain-5 | 3 | 7.86E-03 |
| NP_899630.1 | calcium-dependent secretion activator 1 isoform 3 | 2.99 | 7.94E-03 |
| NP_036467.2 | myosin-If | 2.99 | 8.01E-03 |
| NP_004587.1 | U1 small nuclear ribonucleoprotein A | -2.97 | 8.47E-03 |
| NP_114428.1 | protein ITFG3 | -2.96 | 8.75E-03 |
| NP_065970.2 | alsin isoform 1 | -2.96 | 8.81E-03 |
| NP_003668.2 | density-regulated protein | 2.92 | 9.60E-03 |
| NP_115756.2 | ribosome-releasing factor 2, mitochondrial isoform 1 | -2.88 | 1.07E-02 |
| NP_001864.1 | carboxypeptidase E preproprotein | 2.87 | 1.09E-02 |
| NP_619520.1 | putative ATP-dependent RNA helicase DHX30 isoform 1 | -2.87 | 1.11E-02 |
| NP_001129491.1 | IgG receptor FcRn large subunit p51 precursor | 2.87 | 1.11E-02 |
| NP_060293.2 | dual specificity protein phosphatase 23 | 2.86 | 1.14E-02 |
| NP_066005.2 | protein ALO17 isoform 2 | -2.85 | 1.16E-02 |
| NP_940684.1 | 3-hydroxyacyl-CoA dehydratase 2 | -2.84 | 1.17E-02 |
| NP_002428.1 | protein Mpv17 | 2.84 | 1.19E-02 |
| NP_001129503.2 | ubiquitin-like modifier-activating enzyme ATG7 isoform b | -2.84 | 1.20E-02 |
| NP_987093.1 | COMM domain-containing protein 6 isoform a | -2.81 | 1.27E-02 |
| NP_055315.2 | HIV Tat-specific factor 1 | 2.79 | 1.33E-02 |
| NP_009123.1 | FACT complex subunit SPT16 | -2.72 | 1.58E-02 |
| NP_113657.1 | SH3 domain-binding glutamic acid-rich-like protein 2 | -2.68 | 1.74E-02 |
| NP_078796.2 | hypothetical protein LOC79568 precursor | -2.66 | 1.84E-02 |
| NP_000936.1 | calcineurin subunit B type 1 | -2.66 | 1.84E-02 |
| NP_005196.1 | cytochrome c oxidase subunit 6A2, mitochondrial precursor | -2.65 | 1.88E-02 |
| NP_056288.2 | HEAT repeat-containing protein 5A | -2.63 | 1.97E-02 |
| NP_001120675.1 | PCI domain-containing protein 2 | -2.61 | 2.07E-02 |
| NP_065080.1 | leucine zipper transcription factor-like protein 1 | -2.61 | 2.09E-02 |
| NP_071435.2 | transmembrane BAX inhibitor motif-containing protein 1 | 2.6 | 2.12E-02 |
| NP_003619.2 | plakophilin-4 isoform a | 2.59 | 2.19E-02 |
| NP_004326.1 | bone marrow stromal antigen 2 precursor | -2.57 | 2.29E-02 |
| NP_004100.1 | adrenodoxin, mitochondrial precursor | 2.55 | 2.38E-02 |
| NP_001018098.1 | vacuolar protein sorting-associated protein 13C isoform 2B | 2.53 | 2.51E-02 |
| NP_778228.3 | pleckstrin homology domain-containing family A member 7 | -4.39 | 2.55E-02 |
| NP_001036214.1 | cohesin subunit SA-2 isoform a | 2.52 | 2.55E-02 |
| NP_001011554.1 | solute carrier family 13 member 3 isoform b | -2.51 | 2.61E-02 |
| NP_065691.2 | DCN1-like protein 1 | -2.49 | 2.72E-02 |
| NP_689939.1 | EF-hand domain-containing family member A1 | -2.47 | 2.86E-02 |
| NP_783161.1 | GTPase IMAP family member 8 | 2.47 | 2.89E-02 |
| NP_009210.1 | splicing factor U2AF 65 kDa subunit isoform a | 2.45 | 3.01E-02 |
| NP_000300.1 | protoporphyrinogen oxidase | -2.44 | 3.08E-02 |
| NP_055858.2 | TBC1 domain family member 9B isoform b | -2.43 | 3.10E-02 |
| NP_116103.1 | inverted formin-2 isoform 3 | -2.43 | 3.12E-02 |
| NP_872371.1 | hypothetical protein LOC283991 | 2.43 | 3.13E-02 |
| NP_000396.2 | ganglioside GM2 activator isoform 1 precursor | -2.43 | 3.14E-02 |
| NP_954655.1 | bisphosphoglycerate mutase | -2.42 | 3.21E-02 |
| NP_006263.1 | protein S100-B | -2.4 | 3.32E-02 |
| NP_000345.2 | thyroxine-binding globulin precursor | 2.39 | 3.42E-02 |
| NP_057033.2 | Golgi to ER traffic protein 4 homolog | -2.38 | 3.48E-02 |
| NP_001191742.1 | 5'-nucleotidase isoform 2 preproprotein | 2 | 3.88E-02 |
| NP_115903.1 | microtubule-associated proteins 1A/1B light chain 3A isoform a | 2.33 | 3.93E-02 |
| NP_542198.2 | vacuolar protein sorting-associated protein 41 homolog isoform 2 | 2.32 | 3.96E-02 |
| NP_001123920.1 | hypothetical protein LOC643338 precursor | -2.31 | 4.06E-02 |
| NP_001154992.1 | conserved oligomeric Golgi complex subunit 5 isoform 3 | -2.29 | 4.22E-02 |
| NP_003968.2 | AH receptor-interacting protein | -2.26 | 4.50E-02 |
| NP_612485.2 | magnesium-dependent phosphatase 1 isoform 1 | -2.25 | 4.61E-02 |
| NP_005100.1 | serine/threonine-protein kinase OSR1 | -2.05 | 4.62E-02 |
| NP_006829.1 | methionine aminopeptidase 2 | -2.25 | 4.62E-02 |
| NP_001005333.1 | melanoma-associated antigen D1 isoform a | -2.24 | 4.74E-02 |
| NP_001076581.2 | glycerol-3-phosphate dehydrogenase, mitochondrial precursor | 2.59 | 4.77E-02 |
| NP_006182.2 | proliferation-associated protein 2G4 | 2 | 4.77E-02 |
| NP_055905.2 | neurofascin isoform 4 precursor | -2.23 | 4.83E-02 |

RefSeq: reference sequence; Log2ratio: positive value shows up-regulation, negative value shows down-regulation. P<0.05
