# Supplementary material for: Protein Analysis of Atrial Fibrosis via Label-Free Proteomics in Chronic Atrial Fibrillation Patients with Mitral Valve Disease
Source: PLoS One. 2013 Apr 4;8(4):e60210. doi: 10.1371/journal.pone.0060210 (PMC3617171; doi:10.1371/journal.pone.0060210)
Supplement: Table S2 — 89 differential proteins in RAA of CAF and SR patients. RefSeq: reference sequence; Log2ratio: positive value shows up-regulation, negative value shows down-regulation. P<0.05. (DOC) [file pone.0060210.s005.doc]

**Table S2 89 differential proteins in RAA of CAF and SR patients**

| Refseq | Description | Log2ratio CAF/SR | p-Val CAF/SR |
| --- | --- | --- | --- |
| NP_078970.3 | calpain-7-like protein | 7.51 | 8.13E-11 |
| NP_000584.2 | antigen peptide transporter 1 | -7.14 | 6.20E-10 |
| NP_001139186.1 | SUMO-activating enzyme subunit 1 isoform c | -6.77 | 4.59E-09 |
| NP_004347.1 | CD81 antigen | -6.38 | 3.33E-08 |
| NP_004328.1 | oxidative stress-induced growth inhibitor 2 isoform 2 | -6.36 | 3.65E-08 |
| NP_060264.3 | hypothetical protein LOC54914 | 5.75 | 6.36E-07 |
| NP_065916.2 | probable ATP-dependent RNA helicase DHX36 isoform 1 | -5.71 | 7.61E-07 |
| NP_079517.1 | UBX domain-containing protein 6 isoform 1 | 5.67 | 9.06E-07 |
| NP_061956.2 | tRNA guanosine-2'-O-methyltransferase TRM13 homolog | -5.56 | 1.51E-06 |
| NP_036399.3 | hsp70-binding protein 1 | 5.5 | 1.94E-06 |
| NP_149977.2 | lipase maturation factor 2 | 5.27 | 5.12E-06 |
| NP_000439.1 | V(D)J recombination-activating protein 1 | 5.17 | 7.71E-06 |
| NP_060293.2 | dual specificity protein phosphatase 23 | -5.06 | 1.18E-05 |
| NP_001129101.1 | KLRAQ motif-containing protein 1 isoform 1 | -5.04 | 1.28E-05 |
| NP_005735.2 | E3 ubiquitin-protein ligase ARIH1 | -4.85 | 2.64E-05 |
| NP_653237.1 | mitochondrial intermembrane space import and assembly protein 40 isoform 2 | 4.84 | 2.74E-05 |
| NP_057021.2 | GPI transamidase component PIG-T isoform 1 precursor | 4.84 | 2.76E-05 |
| NP_665734.1 | microsomal glutathione S-transferase 1 | -4.77 | 3.62E-05 |
| NP_006346.1 | tripartite motif-containing protein 38 | 4.75 | 3.88E-05 |
| NP_001185461.1 | oxidation resistance protein 1 isoform 3 | -4.65 | 5.57E-05 |
| NP_114409.2 | plasma alpha-L-fucosidase precursor | 4.56 | 7.84E-05 |
| NP_004242.1 | ras-related protein Rab-9A | -4.4 | 1.38E-04 |
| NP_004108.1 | peptidyl-prolyl cis-trans isomerase FKBP5 isoform 1 | -4.3 | 1.98E-04 |
| NP_000230.1 | lysozyme C precursor | -4.26 | 2.29E-04 |
| NP_057111.1 | beta-lactamase-like protein 2 | -4.25 | 2.30E-04 |
| NP_057270.1 | protein Z-dependent protease inhibitor precursor | 4.25 | 2.30E-04 |
| NP_056009.1 | myosin-Id | 4.14 | 3.32E-04 |
| NP_001135906.1 | bifunctional protein NCOAT isoform b | -4.08 | 4.13E-04 |
| NP_006160.1 | nicotinamide N-methyltransferase | 4.03 | 4.80E-04 |
| NP_001000.2 | 40S ribosomal protein S5 | 3.99 | 5.50E-04 |
| NP_001072987.1 | hypothetical protein LOC283635 isoform 2 | 3.98 | 5.73E-04 |
| NP_001122400.1 | inositol polyphosphate 1-phosphatase | -3.97 | 5.85E-04 |
| NP_444278.1 | 39S ribosomal protein L53, mitochondrial precursor | 3.9 | 7.28E-04 |
| NP_689926.1 | dolichyl-diphosphooligosaccharide--protein glycosyltransferase subunit STT3A | 3.89 | 7.48E-04 |
| NP_112176.1 | ubiquitin-related modifier 1 homolog isoform a | 3.89 | 7.55E-04 |
| NP_003464.1 | signal transducing adapter molecule 1 | 3.58 | 1.07E-03 |
| NP_005598.3 | focal adhesion kinase 1 isoform b | 3.77 | 1.11E-03 |
| NP_002204.2 | integrin beta-5 precursor | -3.72 | 1.28E-03 |
| NP_001017390.1 | sulfotransferase 1A3/1A4 | 4.01 | 1.29E-03 |
| NP_006681.1 | matrix metalloproteinase-24 preproprotein | 3.67 | 1.47E-03 |
| NP_998725.1 | serine/threonine-protein kinase N1 isoform 1 | -3.65 | 1.58E-03 |
| NP_001129503.2 | ubiquitin-like modifier-activating enzyme ATG7 isoform b | -3.61 | 1.78E-03 |
| NP_443107.1 | vacuolar protein sorting-associated protein 26B | 3.61 | 1.79E-03 |
| NP_001121631.1 | vacuolar protein sorting-associated protein 53 homolog isoform 1 | -3.6 | 1.84E-03 |
| NP_004939.1 | desmocollin-1 isoform Dsc1b preproprotein | 3.59 | 1.86E-03 |
| NP_002898.2 | ATP-dependent DNA helicase Q1 | 3.57 | 2.02E-03 |
| NP_065080.1 | leucine zipper transcription factor-like protein 1 | -3.54 | 2.21E-03 |
| NP_055129.2 | probable ATP-dependent RNA helicase DDX58 | -3.53 | 2.27E-03 |
| NP_001352.2 | dihydroorotate dehydrogenase (quinone), mitochondrial precursor | -3.22 | 2.33E-03 |
| NP_570924.2 | receptor-type tyrosine-protein phosphatase S isoform 2 precursor | -3.5 | 2.45E-03 |
| NP_113659.3 | fermitin family homolog 3 short form | 3.29 | 2.49E-03 |
| NP_001029097.1 | nucleolysin TIAR isoform 2 | -3.49 | 2.54E-03 |
| NP_000069.2 | cholesteryl ester transfer protein precursor | 3.44 | 2.88E-03 |
| NP_057103.2 | putative RNA-binding protein Luc7-like 2 | 3.41 | 3.15E-03 |
| NP_071933.2 | ubiquitin-like-conjugating enzyme ATG3 | -3.4 | 3.27E-03 |
| NP_004595.2 | syntaxin-4 | 3.17 | 3.38E-03 |
| NP_065158.3 | hypothetical protein LOC57150 | 3.36 | 3.64E-03 |
| NP_003767.2 | 39S ribosomal protein L40, mitochondrial precursor | 3.27 | 4.67E-03 |
| NP_001153778.1 | synaptojanin-1 isoform d | -3.21 | 5.46E-03 |
| NP_003144.3 | signal transducer and activator of transcription 6 isoform 1 | 3.2 | 5.52E-03 |
| NP_005773.3 | THO complex subunit 4 | -3.17 | 6.02E-03 |
| NP_006247.1 | serine/threonine-protein kinase N2 | 3.17 | 6.08E-03 |
| NP_005073.2 | E3 ubiquitin/ISG15 ligase TRIM25 | -3.11 | 7.02E-03 |
| NP_803877.2 | uridine diphosphate glucose pyrophosphatase | -3.09 | 7.39E-03 |
| XP_001126659.1 | PREDICTED: hypothetical protein LOC728026 | 3.08 | 7.58E-03 |
| NP_001736.1 | calcium signal-modulating cyclophilin ligand | 3.07 | 7.81E-03 |
| NP_055870.2 | switch-associated protein 70 | 3.05 | 8.32E-03 |
| NP_067000.1 | U6 snRNA-associated Sm-like protein LSm2 | 3.04 | 8.51E-03 |
| NP_003860.2 | cocaine esterase isoform 1 | 2.88 | 8.69E-03 |
| NP_000146.2 | galactose-1-phosphate uridylyltransferase | -2.87 | 8.71E-03 |
| NP_001182465.1 | charged multivesicular body protein 5 isoform 2 | 3 | 9.30E-03 |
| NP_003293.2 | thyroid receptor-interacting protein 6 | -2.97 | 1.01E-02 |
| NP_000101.2 | dihydropyrimidine dehydrogenase [NADP+] isoform 1 | 3.07 | 1.01E-02 |
| NP_009166.2 | exportin-T | 3.55 | 1.09E-02 |
| NP_004138.1 | developmentally-regulated GTP-binding protein 1 | -2.94 | 1.10E-02 |
| NP_689979.1 | hypothetical protein LOC254863 precursor | 2.99 | 1.12E-02 |
| NP_057292.1 | vacuolar protein sorting-associated protein 28 homolog isoform 1 | 2.88 | 1.27E-02 |
| NP_008835.5 | DNA-dependent protein kinase catalytic subunit isoform 1 | 2.77 | 1.31E-02 |
| NP_001158503.1 | thymidylate kinase isoform 2 | -2.86 | 1.34E-02 |
| NP_003681.1 | interferon-inducible double stranded RNA-dependent protein kinase activator A isoform 1 | 2.85 | 1.36E-02 |
| NP_000705.2 | translocator protein isoform PBR | 2.85 | 1.36E-02 |
| NP_775907.4 | WD repeat-containing protein 62 isoform 2 | 2.79 | 1.58E-02 |
| NP_002007.1 | filaggrin | -2.78 | 1.62E-02 |
| NP_001139731.1 | phosphatidylinositol-5-phosphate 4-kinase type-2 gamma isoform b | 2.77 | 1.64E-02 |
| NP_003968.2 | AH receptor-interacting protein | -2.75 | 1.73E-02 |
| NP_037374.1 | transcription factor IIB | 2.75 | 1.74E-02 |
| NP_038472.2 | ubiquilin-2 | 2.74 | 1.88E-02 |
| NP_001165084.1 | prolyl endopeptidase-like isoform 4 | 2.83 | 2.01E-02 |
| NP_003720.1 | RNA 3'-terminal phosphate cyclase isoform b | -2.37 | 2.14E-02 |
| NP_037368.1 | ataxin-10 isoform 1 | 2.64 | 2.23E-02 |
| NP_291028.3 | TP53-regulating kinase | 2.64 | 2.24E-02 |
| NP_009016.1 | follistatin-related protein 1 precursor | 2.61 | 2.38E-02 |
| NP_006054.2 | kelch repeat and BTB domain-containing protein 10 | 2.55 | 2.40E-02 |
| NP_005898.2 | mannosyl-oligosaccharide 1,2-alpha-mannosidase IA | -2.58 | 2.56E-02 |
| NP_036467.2 | myosin-If | 2.58 | 2.57E-02 |
| NP_055597.1 | armadillo repeat-containing X-linked protein 2 | 2.57 | 2.59E-02 |
| NP_001275.1 | AP-3 complex subunit sigma-1 | -2.56 | 2.64E-02 |
| NP_057543.2 | plasma cell-induced resident endoplasmic reticulum protein precursor | 2.54 | 2.76E-02 |
| NP_001005333.1 | melanoma-associated antigen D1 isoform a | -2.53 | 2.86E-02 |
| NP_001036189.1 | poliovirus receptor-related protein 2 isoform delta precursor | 2.52 | 2.91E-02 |
| NP_060439.2 | protein IWS1 homolog | -2.51 | 2.97E-02 |
| NP_775925.1 | AER61 glycosyltransferase | 2.5 | 3.05E-02 |
| NP_001014796.1 | discoidin domain-containing receptor 2 precursor | -2.48 | 3.20E-02 |
| NP_055858.2 | TBC1 domain family member 9B isoform b | 2.47 | 3.22E-02 |
| NP_055439.1 | protein S100-A6 | 2.47 | 3.28E-02 |
| NP_006792.1 | ER lumen protein retaining receptor 1 | -2.46 | 3.34E-02 |
| NP_001193940.1 | ciliary neurotrophic factor receptor subunit alpha preproprotein | 2.45 | 3.38E-02 |
| NP_061932.1 | mitochondrial import receptor subunit TOM7 homolog | 2.45 | 3.40E-02 |
| NP_542193.3 | BRI3-binding protein | -2.44 | 3.45E-02 |
| NP_001171691.1 | clathrin light chain A isoform f | 2.66 | 3.52E-02 |
| NP_002090.4 | glycophorin-A precursor | 2.42 | 3.60E-02 |
| NP_000496.2 | coagulation factor XII precursor | 2.42 | 3.60E-02 |
| NP_009123.1 | FACT complex subunit SPT16 | -2.41 | 3.67E-02 |
| NP_951008.1 | UPF0636 protein C4orf41 isoform b | 2.36 | 4.10E-02 |
| NP_064506.3 | UDP-glucose:glycoprotein glucosyltransferase 2 precursor | -2.35 | 4.16E-02 |
| NP_009201.2 | peptidyl-prolyl cis-trans isomerase FKBP9 precursor | 2.7 | 4.16E-02 |
| NP_003668.2 | density-regulated protein | -2.35 | 4.21E-02 |
| NP_001185987.1 | C15orf38-AP3S2 fusion protein | -2.34 | 4.25E-02 |
| NP_065142.2 | 39S ribosomal protein L47, mitochondrial isoform a | -2.32 | 4.41E-02 |
| NP_004263.1 | homer protein homolog 1 | -2.32 | 4.47E-02 |
| NP_055419.1 | tax1-binding protein 3 isoform 1 | -2.28 | 4.87E-02 |

RefSeq: reference sequence; Log2ratio: positive value shows up-regulation, negative value shows down-regulation. P<0.05
