# Supplementary material for: Protein Analysis of Atrial Fibrosis via Label-Free Proteomics in Chronic Atrial Fibrillation Patients with Mitral Valve Disease
Source: PLoS One. 2013 Apr 4;8(4):e60210. doi: 10.1371/journal.pone.0060210 (PMC3617171; doi:10.1371/journal.pone.0060210)
Supplement: Table S3 — Enrichment p-valve in functions and pathways of 223 differentially expressed proteins identified by Label-free proteomic (p<0.05). (DOC) [file pone.0060210.s006.doc]

**Table S3** Enrichment p-valve in functions and pathways of 223 differentially expressed proteins identified by Label-free proteomic (p<0.05)

| Enrichment in functions and pathways p Value |
| --- |
| Jak-STAT signaling pathway 0.006  Cell proliferation 0.01  Response to stress 0.01  Immune response 0.01  Cell cycle 0.02  Focal adhesion 0.02  Apoptosis 0.04  Adherens junction 0.05 |
